# Supplementary material for: Participatory and Spatial Analyses of Environmental Justice Communities’ Concerns about a Proposed Storm Surge and Flood Protection Seawall
Source: Int J Environ Res Public Health. 2022 Sep 6;19(18):11192. doi: 10.3390/ijerph191811192 (PMC9517186; doi:10.3390/ijerph191811192)
Supplement: Supplementary file 1 [file ijerph-19-11192-s001.zip › ijerph-1879694-supplementary.pdf]

SUPPLEMENTAL MATERIAL

Table S1. Dates, locations, stakeholders present, purpose, and subject matter of the community meetings where the first author attended and recorded field observations.

Date, Time	Location	Stakeholders present (approx. number of attendees)	Purpose of meeting	Subject matter discussed
June 25-26, 2021	Perry Webb Community Center (Accabee)	Residents and leaders from LAMC communities (4)<br>Residents and leaders from non-LAMC communities (21)<br>LAMC/CCRAB (3)<br>University of South Carolina (2)<br>College of Charleston (2)<br>S.C. Dept. of Health and Environmental Control (5)	EJ Strong workshop 1	
July 23, 2021, 1PM	Freddie Whaley Community Center (Rosemont)	USACE (2)<br>City of Charleston (1)<br>LAMC/CCRAB (3)<br>Coastal Conservation League (2)	USACE early engagement in Rosemont, walking tour	USACE seawall, flooding issues in Rosemont
October 1, 2021, 4PM	Rosemont	LAMC/CCRAB (2)<br>University of South Carolina (1)<br>Rosemont residents (15)	Canvassing in Rosemont	Student research, seawall, flooding
October 23, 2021, 2PM	Freddie Whaley Community Center (Rosemont)	USACE (3)<br>City of Charleston (2)<br>Groundswell (2)<br>Rosemont Neighborhood Association/Rosemont residents (25)<br>LAMC/CCRAB (3)	USACE and City of Charleston discussing nonstructural mitigation and the seawall with Rosemont residents	USACE seawall, nonstructural mitigation, flooding issues in Rosemont
November 10, 2021, 12PM	Zoom	LAMC/CCRAB (2)<br>Rosemont Neighborhood Association/Rosemont residents (8)<br>Coastal Conservation League (CCL) (2)<br>Southern Environmental Law Center (SELC) (2)	CCL engaging with Rosemont to discuss the October 23 USACE meeting	USACE seawall
December 4, 2021, 10AM	1998 Hugo Ave, North Charleston (Union Heights)	LAMC/CCRAB (5)<br>Union Heights resident (1)	Photovoice training held by CCRAB	Flooding in the LAMC communities, USACE seawall, issues with road maintenance in LAMC communities

Table S2. Data layers incorporated into the EJ profile, in alphabetical order.

**Data Layer**	**Description**	**Source**
Community boundaries	Polygon boundaries of the Charleston Neck communities, including Accabee, Chicora/Cherokee, Liberty Hill, Union Heights, Windsor Place, Rosemont, Garden Hill, and Five Mile	LCRT [18] NEPA report, conversations with residents
Digital elevation model (DEM)	Raster layer depicting elevation in the Charleston area	CofC Lowcountry Hazards Center
Digital surface model (DSM) of Rosemont	Raster layer depicting surface elevation in the Rosemont community	Drone
Flood projections from the Dutch Dialogues	Dutch Dialogues-produced locations of flood risk, modified from DEM	CofC Lowcountry Hazards Center
Flood risk locations	Parts of the Charleston Neck area that have flooded or flood frequently	Interview results, conversations with residents
Important places	Polygons/points/lines depicting critical assets in the Charleston Neck. Could include grocery stores, roads, churches, gathering places, parks, community centers, bus stops, etc.	Interview results, conversations with residents
Industrial facilities	Locations of industrial facilities that have experienced hazardous releases	EPA's Toxic Release Inventory (TRI)
Land cover	Developed areas, impervious surfaces, saltmarsh, etc.,	NLCD 2019 [65]
Living shorelines	Polylines depicting the locations of proposed living shorelines/oyster beds	Digitized from USACE documents/publications
NHC storm surge hazard maps	Depict storm surge potential in a high tide scenario using the Sea, Lake, and Overland Surges from Hurricanes (SLOSH) model	NOAA National Hurricane Center (NHC)/CofC Lowcountry Hazards Center
Nonstructural mitigation	Polygons depicting the locations of proposed nonstructural mitigation (floodproofing, elevating homes)	Digitized from USACE documents/publications
Pumping stations	Polygons depicting the locations of proposed pumping stations	Digitized from USACE documents/publications
Sea level rise projections	Raster layers from NOAA depicting sea level rise inundation in 1ft increments between 0ft and 10ft	NOAA
Seawall	Polyline depicting the location of the proposed seawall, including specifications on the type of seawall (T-wall vs. combo wall)	Digitized from USACE documents/publications
Streets	Polylines depicting locations of streets in the Charleston Neck	SCDOT [66]

Copy of Blank Consent Form

Consent to Participate in Research

You are invited to participate in a research study. This research will be conducted by Judith Taylor, a graduate student in the College of Charleston's Environmental and Sustainability Studies program, under the supervision of Dr. Paul Sandifer from CofC's Center for Coastal Environmental and Human Health and in collaboration with the Lowcountry Alliance for Model Communities (LAMC), and is designed to investigate any potential positive or negative effects of the proposed downtown Battery seawall on upper peninsula neck communities.

Participation in this study will require about 15-30 minutes for an interview. As a participant in this research, you will be asked to contribute to the study by offering insight into your community, flood risk in your community/the City of Charleston, and the seawall project, and offering suggestions for data or topics to incorporate into the study. I will use your suggestions and knowledge to guide research into impacts of the seawall on neck communities, and will hold optional public meetings to present findings and collaboratively discuss next steps. I will be recording audio from the interviews in order to better collect information to use in the study.

I will keep your information strictly confidential. However, If you are willing to permit me to quote you in the report of my research, please check the item just above the signature line. You will be given an opportunity to review the section of my report in which your quote appears before completion of my research.

> The audio tapes from the interviews will be kept for the duration of the study, or a maximum of a year after they are collected. They will be kept in a password protected folder on my personal laptop for use in this research only. After the conclusion of the study, data will be destroyed, or if requested by the participant, archived with LAMC for future reference.

Although it is not anticipated that you will benefit directly through your involvement in this study, this research is expected to benefit both members of neck communities and seawall project stakeholders by compiling a collaborative environmental justice perspective**.**

I know of no risk or discomfort associated with this research.

Your participation is completely voluntary, and you may discontinue participation at any time. Your consent is voluntary and may be withdrawn at any time.

Your personal data collected for this research will be stored for the duration of this study, or a maximum of one year after it is collected.

If you have any questions concerning this research study please contact Judith Taylor at 203-305-8556 or taylorj4@g.cofc.edu or my faculty advisor at 843-709-4333 or sandiferpa@cofc.edu. You may also contact Research Protections & Compliance on the Office of Research and Grants Administration, at 843-953-5885 or e-mail compliance@cofc.edu if you have questions or concerns about research review at the College of Charleston or your rights as a research participant. You will be given a copy of this form to keep.

This research has been reviewed by the Human Research Protections Program at the College of Charleston and covers all relevant requirements of the EU General Data Protection Regulations.

The information in this consent form has been explained to me, I have been given the opportunity to ask questions, and I certify that I am at least 18 years old.

Regarding storage of audio recordings after the conclusion of the study,
I request my data be ___ destroyed ___ archived with LAMC

In any reports or publications which result from this research,
I permit you to quote me ___ no ___ yes

____
Printed Name of Participant

____ ________________
Signature of Participant Date

____ ________________
Signature of Person Obtaining Consent Date

If you would like to receive a copy of the results of this study, please print your contact information (mailing address or e-mail):

SUPPLEMENTAL MATERIAL

Story Map

Link: https://storymaps.arcgis.com/stories/850419153fb7414391c692fc3a0a794f

Story Map Screenshots

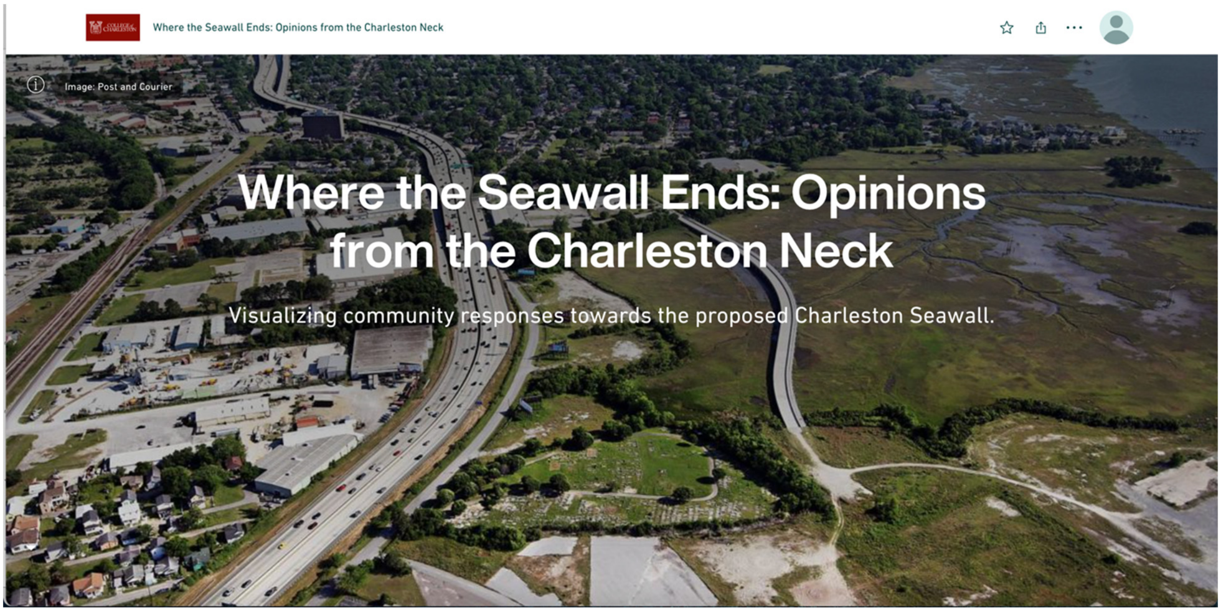

On this page... | EJ Profile... | Results from outreach... | Interactive map contents... | Interactive map... | Contact Information...

On this page...

- EJ Profile on the communities in the neck
- Results from community outreach
- How to use the interactive map
- Data descriptions for interactive map
- Interactive map
- Map highlights
- Contact information

Use the tabs at the top to quickly navigate to these sections, or scroll through for the full report.

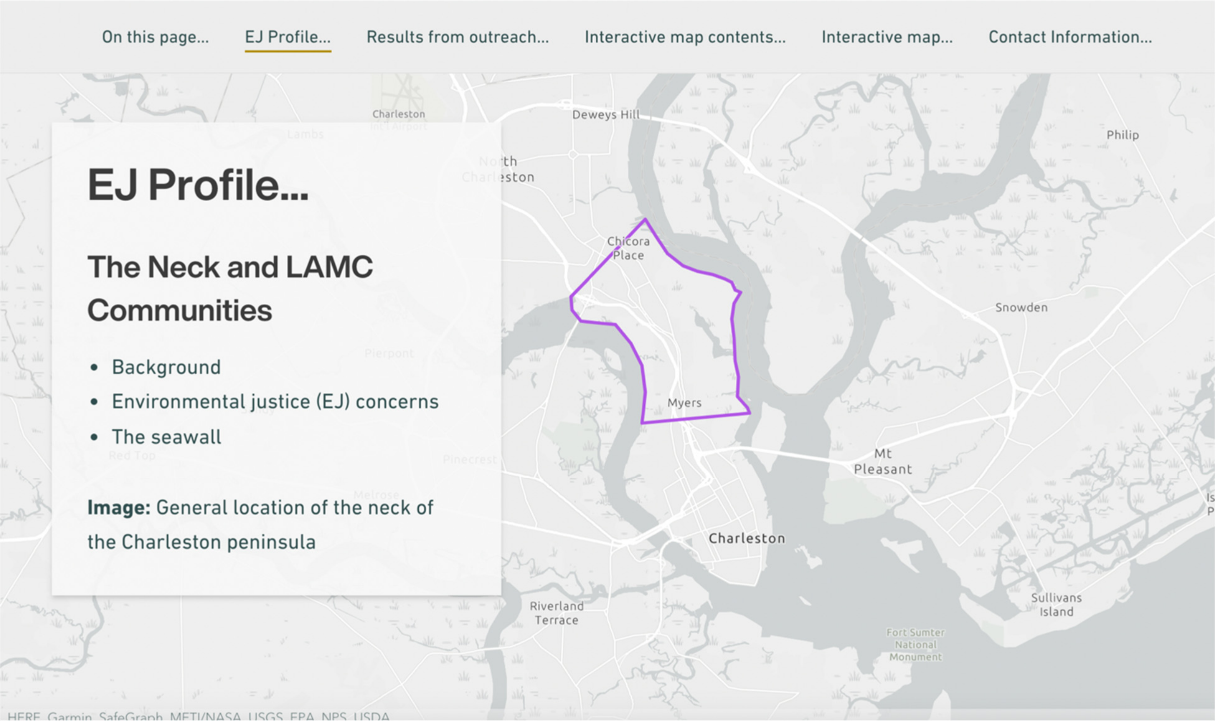
On this page...
EJ Profile...
Results from outreach...
Interactive map contents...
Interactive map...
Contact Information...
EJ Profile...
The Neck and LAMC Communities
• Background
• Environmental justice (EJ) concerns
• The seawall
Image: General location of the neck of the Charleston peninsula
Deweys Hill
Chicora Place
Myers
Philip
Snowden
Mt Pleasant
Charleston
Riverland Terrace
Sullivans Island
Fort Sumter National Monument
HERE, Garmin, SafeGraph, METI/NASA, USGS, EPA, NPS, USDA

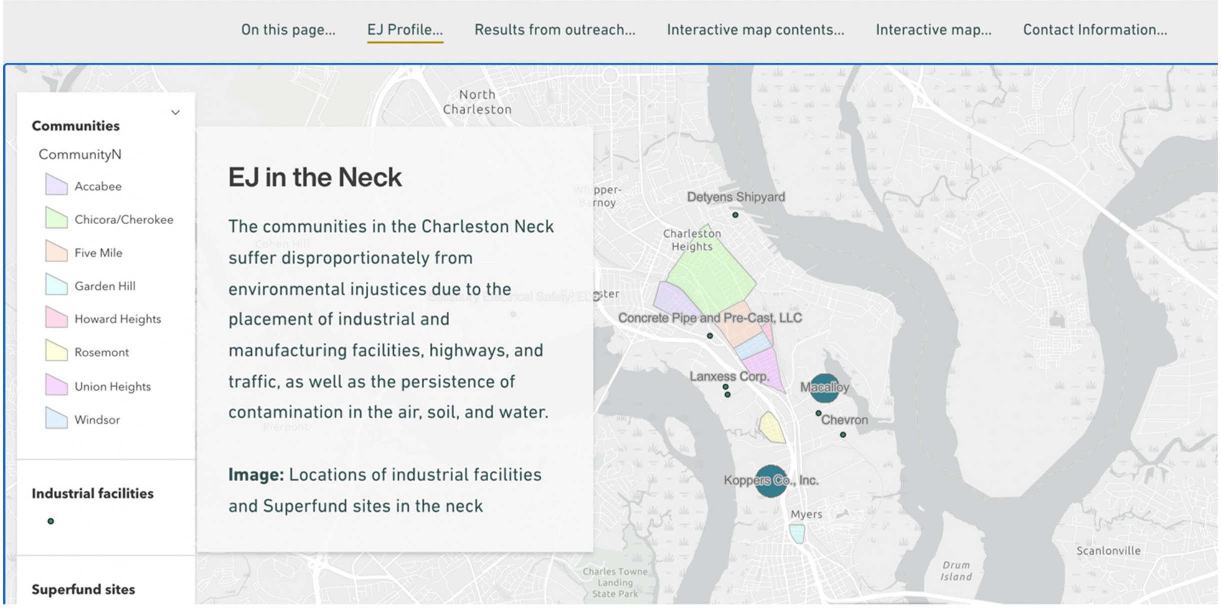
On this page...
EJ Profile...
Results from outreach...
Interactive map contents...
Interactive map...
Contact Information...
Communities
CommunityN
Accabee
Chicora/Cherokee
Five Mile
Garden Hill
Howard Heights
Rosemont
Union Heights
Windsor
Industrial facilities
Superfund sites
EJ in the Neck
The communities in the Charleston Neck suffer disproportionately from environmental injustices due to the placement of industrial and manufacturing facilities, highways, and traffic, as well as the persistence of contamination in the air, soil, and water.
Image: Locations of industrial facilities and Superfund sites in the neck
North Charleston
Detyens Shipyard
Charleston Heights
Concrete Pipe and Pre-Cast, LLC
Lanxess Corp.
Macalloy
Chevron
Koppers Co., Inc.
Myers
Charles Towne Landing State Park
Drum Island
Scanlonville

On this page... EJ Profile... Results from outreach... Interactive map contents... Interactive map... Contact Information...

LAMC

The Lowcountry Alliance for Model Communities (LAMC) is a nonprofit that supports neck community work in education, affordable housing, economic development, and environmental justice, alongside its research group, the Charleston Community Research to Action Board (CCRAB).

LAMC's comments about the need to address environmental justice impacts of the wall were part of the reason the U.S. Army Corps of Engineers (USACE) drafted an Environmental Impact Statement (EIS) in addition to the original Environmental Assessment (EA). LAMC [illegible]

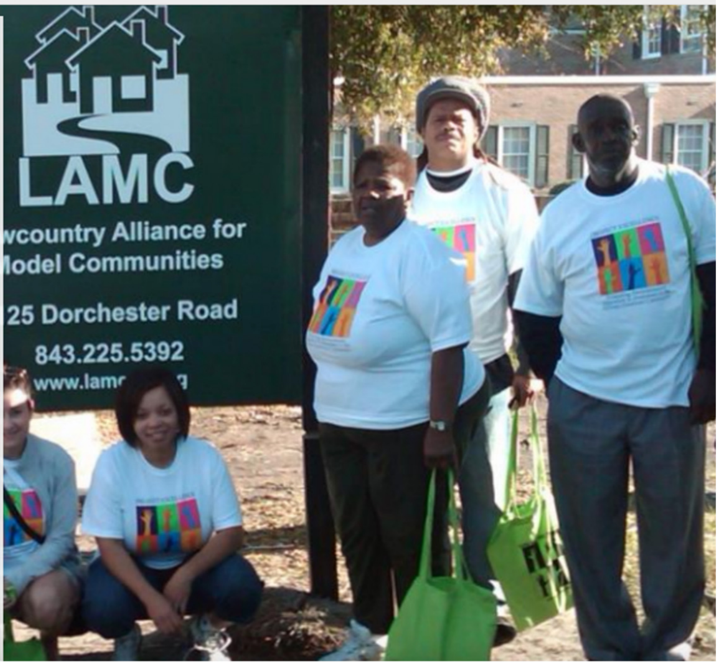

On this page... EJ Profile... Results from outreach... Interactive map contents... Interactive map... Contact Information...

The Seawall - Timeline

Oct 2018 | USACE peninsula study is announced

Apr 2020 | USACE releases draft Feasibility Report and Environmental Assessment (EA)

Apr-Jun 2020 | 60-day public comment period for EA

Mar 2021 | USACE announces they will complete an Environmental Impact Statement (EIS)

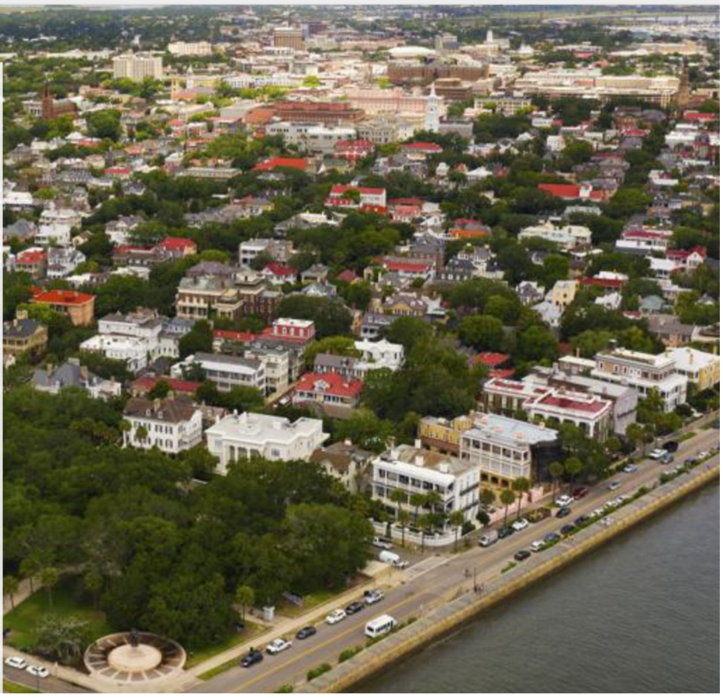

On this page... EJ Profile... Results from outreach... Interactive map contents... Interactive map... Contact Information...

The Seawall - Design

The wall will stand 12-feet tall and will wrap around the peninsula and cut off near Heriot Street. Natural shoreline features have been added along the Ashley River to reduce impacts to the marsh.

Non-structural flood mitigation is being recommended for Rosemont in place of a wall. This will include elevating homes that can be raised, and floodproofing homes that can't be raised.

Image: the most recent design of the wall

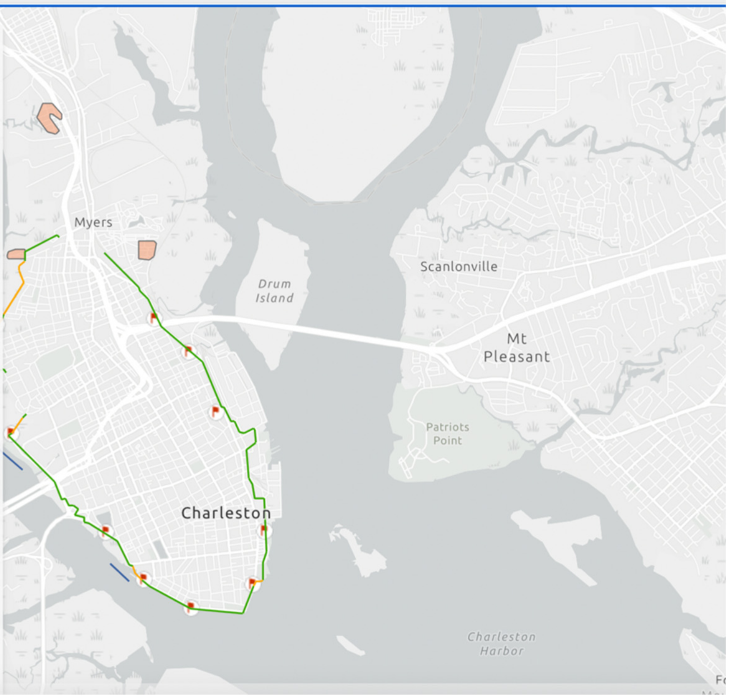

On this page... EJ Profile... Results from outreach... Interactive map contents... Interactive map... Contact Information...

While the Corps has said these communities are at a higher elevation than downtown Charleston and therefore do not need a wall, there are significant parts of the neck that are below the wall's elevation of 12-feet.

This has led to concerns about flooding continuing and even worsening if a wall is constructed downtown.

The Corps and the City of Charleston have engaged with the Rosemont community about implementing nonstructural flood mitigation, but the other neck communities that lie in North

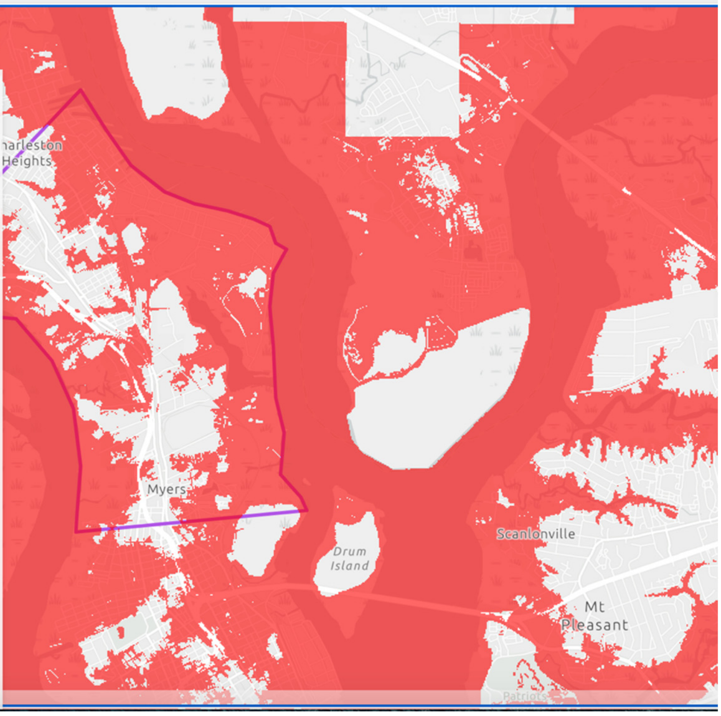

On this page... EJ Profile... Results from outreach... Interactive map contents... Interactive map... Contact Information...

Results from outreach...

This study sought to collect and explore neck residents' concerns about the seawall and other issues facing their communities.

Information was collected through interviews and conversations with residents and leaders, as well as observations from community meetings between Summer 2021 and Spring 2022.

The following contains quotes and results from these interviews, conversations, and meetings on a variety of topics, including people's communities, experiences with flooding, opinions about the seawall, and suggestions for this report.

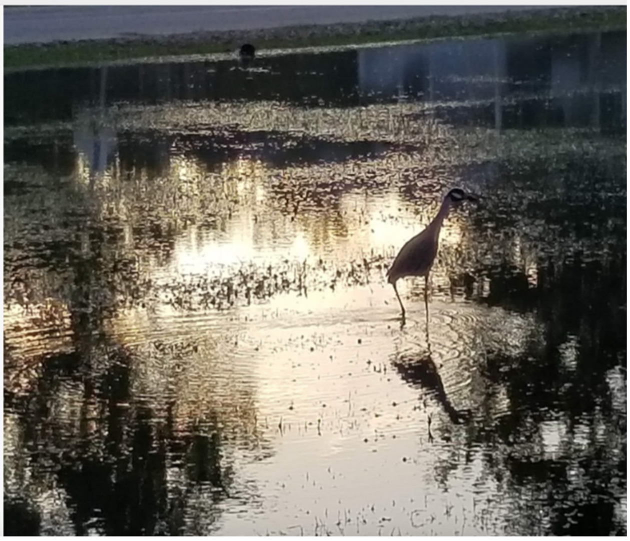

Image: Flooding in Rosemont taken by resident

On this page... EJ Profile... Results from outreach... Interactive map contents... Interactive map... Contact Information...

Tell me about your community.

"It's a good close group of people from generation to generation...they look out for each other there as all communities do. There's a sense of taking care of those who are residents there."

"...there's a lot of historical values about Rosemont. It's one of the first black African American neighborhoods established after the reconstruction era or after the Civil War, so the land, the people, are all very important in the history of the City of Charleston."

"...this'll always be home. And I'm always gonna care about what happens."

"If these areas didn't exist, these communities didn't exist, I wouldn't be who I am."

"I just love it because it's like in the center of everything, it takes just a matter of minutes to get anywhere that you need really, so the convenience is great."

"I see it as a badge of honor to be able to be a resident there, I see it as a badge of honor for me to live there and raise my children there."

On this page... EJ Profile... Results from outreach... Interactive map contents... Interactive map... Contact Information...

Can you describe your community in three words?

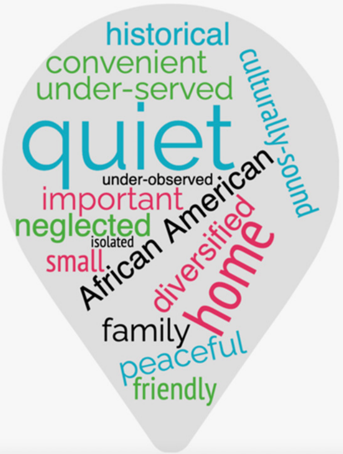

Has your community changed since you've lived here?

"It used to be a really beautiful community growing up, but it seems like as the years have gone by the City of Charleston has like, they don't keep up with the community and our needs like we need them to, I feel like we are a forgotten community..."

"...the diversity it's changing, before it was like an all African American community and now you see other ethnicities moving in the neighborhood."

"...there's a lot more traffic and a lot more development coming around us in the area..."

"I want to grow a garden and we've thought about it, but with that chemical plant so nearby I've always had concerns about growing food and actually eating it here in this neighborhood."

"It's one of those communities that's culturally underserved and the disparities is visible in the housing, the jobs..."

"...Rosemont is Rosemont and that isolation exists and all the other disparities, it's a game of survival."

"...there were a lot of things this community had...like a little grocery store, we had cleaners, we had all kinda things that people had businesses and stuff in this community, you know all that changed."

"...people started moving out and people came in renting...some people if they're renting it's not theirs so they don't care about it..."

On this page... EJ Profile... Results from outreach... Interactive map contents... Interactive map... Contact Information...

Does your community experience flooding?

"July 20th 2019, we had a really bad flood around here. That's where we had cars floating and we found it could be traced back to the materials from the highway construction. It got really bad, Bertha's restaurant right here on Meeting Street they had to replace all of their steam tables, refrigeration equipment cause everything got flooded."

"...my dad used to have to pick me up to get to the door to put me in the car, we always had boots..."

"...a gentleman that lives down one of the streets that sits on the marsh says, 'man, they paved the streets and I'm so grateful for that, but before the paving, water would roll down because the street inclines that way and it would go right to the marsh. Now because of the paving in the street and the spacing between the street and the sidewalk, there's this little gully that's filled and the water spills over the sidewalk over to my property...' "

On this page... EJ Profile... Results from outreach... Interactive map contents... Interactive map... Contact Information...

Have you noticed any changes in the flooding?

"...these [were] unincorporated areas of the county, so they weren't part of any municipality and they were underserved areas. So nobody had any maintenance plan for their storm drainage, upkeep for their storm drainage...it nearly did not exist and it wasn't well designed with what did exist, so when they were annexed into the two cities that was never considered. So the same drainage problems and flooding problems that existed then, still exist now."

"...Rosemont, we know that it floods more frequently now because the water runs off of the interstate."

"When I was a kid in the city, it didn't do much good, but one of the things they did about once a quarter is they'd open the drains on the side of the street, you know next to the sidewalk...they'd reach into the drains and they'd pull stuff up out of the drains and make sure the drains were clear. That kinda thing doesn't happen anymore."

On this page... EJ Profile... Results from outreach... Interactive map contents... Interactive map... Contact Information...

Where have you seen flooding?

Image: Locations of places participants have seen flooding, including the shipyard, Riverview Ave, Beech Ave, Echo Ave, Irving Ave, and Little Ave in North Charleston, and along the marsh-side of Rosemont, Austin Ave, Doscher Ave, and Food Lion on King St in Charleston (can scroll and zoom into the map on the right).

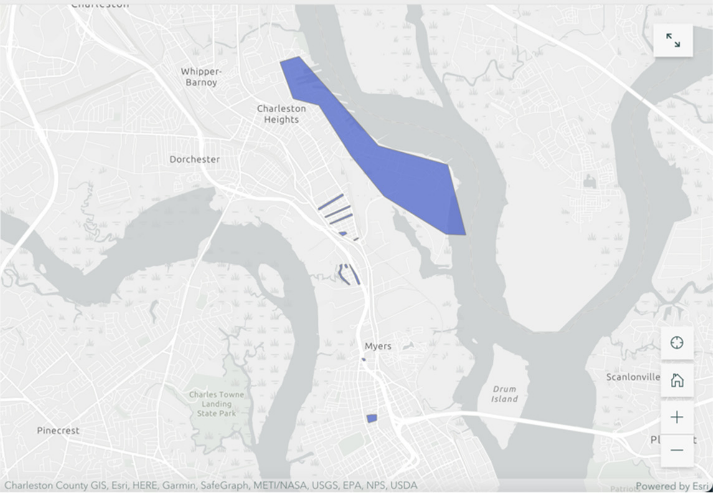

On this page... EJ Profile... Results from outreach... Interactive map contents... Interactive map... Contact Information...

What does a resilient Charleston look like to you?

"When in '89 when Hurricane Hugo hit here, this city was tore up, but we came together as a city, as a community, and all the communities came together and pitched in and removed all that debris and all that waste...it didn't take long for us to begin to look like nothing happened."

"I would like to see more green spaces, drainage improvement back here..."

"...we have to unify Charleston, we need to put away our differences and really focus on making sure Charleston that is here for the next hundred years...that's how we can build a better, resilient Charleston in my eyes..."

"A neighborhood where the people have a place to congregate like a park or something with activities...a clean space, like I said where somewhere people can go to relax."

On this page... EJ Profile... Results from outreach... Interactive map contents... Interactive map... Contact Information...

Can you think of ways your community could benefit from the seawall?

"...the idea of preserving this is commendable, it has to be done...now rather than later, and I applaud the mayor and the council for having the insight to look at, we can start doing something now instead of waiting till it jumps up on us...We need to preserve this history, we need to preserve this culture, we need to look at keeping the city functional because of everything else that has a hand out to this city...look at the financial impact of what would happen if Charleston was to go under..."

"...from a project as a whole, that would give us the opportunity to protect ourselves a lot better..."

"I really just don't think it's gonna benefit us at all, I won't mind if it's gonna benefit us, it's not, it's gonna take Rosemont down in so many different ways."

"I don't see any benefit to it at all, actually I feel it's gonna be a disaster for the neighborhood."

"I think that [elevating homes] would be a good thing for those who don't have disabilities."

On this page... EJ Profile... Results from outreach... Interactive map contents... Interactive map... Contact Information...

Do you have any concerns about the seawall?

"...that seawall is going to be effective for probably the next twenty years. In twenty years, it won't be effective anymore and the damage that occurs to the surrounding areas as a result of the erosion that occurs because of the seawall, is gonna be irreparable. 'Cause once the sand washes away to the ocean, that's bad."

"If they could successfully elevate the homes then that would be great, I'm all for it...I just really don't see it happening, I think the Corps will probably say in the end that it's too expensive to do."

"I'm afraid with that wall, that water is gonna come here."

"I think we pose a really strong issue for our seniors who have bad knees bad hips, even though 3 feet doesn't sound like a lot, that's an extra 2 to 3 steps that someone has to climb in order to get in and out of their house..."

"...we don't want to leave anyone behind so let's not leave Rosemont behind, that's where my concerns are, that's where the residents' concerns are..."

"I believe that our taxes are going to be going up here soon, and those who have fixed incomes, how do you continue to...be able to live in an area like where we're living in, so I think that will be a very popular neighborhood in the next 5 to 10 years. Gentrification is happening everywhere..."

On this page... EJ Profile... Results from outreach... Interactive map contents... Interactive map... Contact Information...

Do you have any questions about the seawall?

"...if you're trying to keep the water out of this area then where is the water going to go? Is it gonna push towards our area, and where is that gonna leave us? You're gonna leave our homes to flood?"

"...what is this wall going to look like, and how are we supposed to get out of this wall, if it only has 4 exits..."

"I would like to know what the material is made up of, how long they think that the wall is going to be able to stand without I guess having renovations done..."

"...what happens to this walled city if two dams in the Santee Cooper system break. How much water is going to go down the Cooper River and how much is going to come on to the peninsula."

"...I would like to see reports from Landnex I think it is, and how they are looking to potentially protect their business...because those business are around where we are, so I would like to see what their plan is for flooding and to protect them from the seawall..."

"...what they have done to inform all the people, all the neighborhoods, have they actually touched bases other than on the news to make sure that all the residents know what's going on..."

On this page... EJ Profile... Results from outreach... Interactive map contents... Interactive map... Contact Information...

What would you like to see in your community?

"I think we need more street lights, we definitely need maybe like a bus shelter, cause we got the sign, but we ain't got no bus shelter."

"...because we have access here to [I-26], they take shortcuts through [Garden Hill]...there's a couple of children around here and that's dangerous, the kids could be playing and it's not like we have a park or anything, they just play under the bridge or in front of their house, we don't have sidewalks."

"...we don't even have a drainage system on the sidewalks or our roads, so...there's no place for the water to go except for downhill. I think that if we got drains that would help us a whole lot."

"It would be a lot better if there were just trees and not the intrusive highways and trucks and other traffic that comes with it, and the pollution that they bring with them."

"...we need them to fix the drainage system around here, a couple of the streets and especially under this bridge, we can't even see the drain anymore, it's filled with trash..."

On this page... EJ Profile... Results from outreach... Interactive map contents... Interactive map... Contact Information...

Suggestions for the city and the Army Corps.

"Include us now in the dynamics of what has to be done, to look at how to get this project going off the ground and start it up, and include all segments of the city of Charleston..."

"...look at Charleston realistically before you develop, before you build."

"...that's something that the City of Charleston needs to take into consideration for us, is how our drainage system is working and is there a proper drainage system for us."

"...I would like to see...a real opportunity for us to sit down with our Councilmen, not just Mitchell but our whole entire City Council, and see why the Army Corps of Engineers...have discluded Rosemont from the report, from the seawall, and it's kinda just have the opportunity to have an open discussion."

"Have some vision to say, how is it going to impact the farthest reaches of the Ashley River all the way to Baking Bridge Road in Summerville and beyond."

On this page... EJ Profile... Results from outreach... Interactive map contents... Interactive map... Contact Information...

Suggestions for this report.

"...express our sense of advocacy for betterment of the community, no more no less, our just due. And in saying that help us find the resources so that our struggle is one we can overcome and be representative of the city, we're Charleston, and as such help us live to those standards, include us in the well-being."

"...using GPS and satellite images so that you can make comparisons of that 8 mile thing compared to the width of the harbor."

"...photos of the flooding."

"...I was gonna try to find out how the elevations and different areas were, cause I know that makes a difference even for the flood insurance and everything..."

"...aerial photos even with like Google Earth of the roads that are back here with the road project when they started it like 2016, 2017...the road is so messed up back there and they have yet to fix it."

On this page... EJ Profile... Results from outreach... Interactive map contents... Interactive map... Contact Information...

Discussions about community advocacy.

"...because we're strong minded about trying to hold onto our community like I said that's our heritage, I think that's what makes us strong, stronger as a community, and we have people who wants to come back, you know, and they would like to see this community grow and strive, 'cause we got a lot of memories, so I think as a team we can resist anything."

"I hope that with each passing generation some sense of newness will come about, with different social groups that will come in and offer some sense of guidance to a better place that will take hold and start elevating, especially the youth, making quality of life better for them. With the community council, it's about trying to be informed about the different things the city is implementing or wants to implement, and how we can get into that and be a part of it and garner some of this sense of opportunity to help us grow..."

"I wanna be able to take on that identity and to continue to have that strength...it's important to me to preserve [the community], it's important for me to have that opportunity for the next generation to be exposed to that as well."

Opinions about the seawall fell into 5 buckets:

- Potential benefits
- Concerns
- Questions
- Suggestions
- Perceptions of resilience

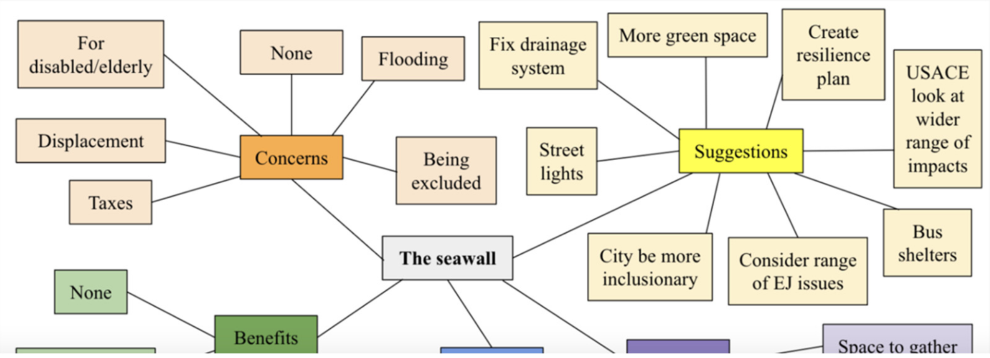

On this page... EJ Profile... Results from outreach... Interactive map contents... Interactive map... Contact Information...

Participants from Rosemont supplied photo evidence of flooding in their community:

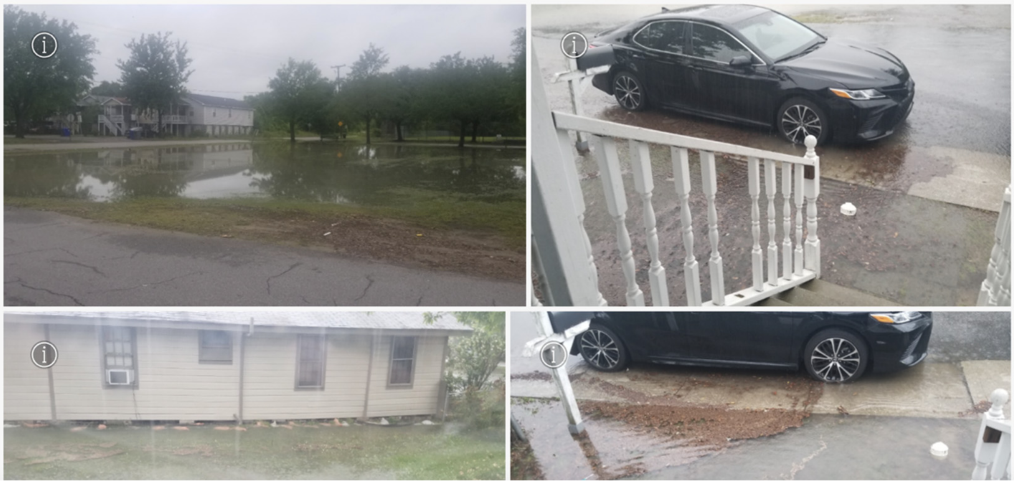

I took the following pictures of flooding under the I-26 bridge in Charleston along Courtland Ave, next to the Garden Hill Community, an area that floods often according to a resident:

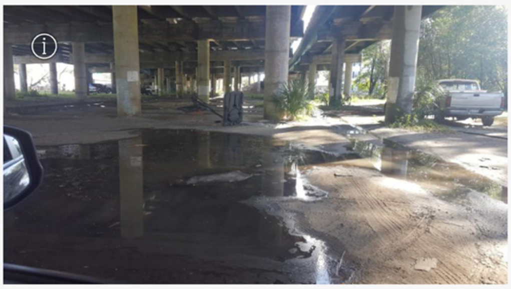

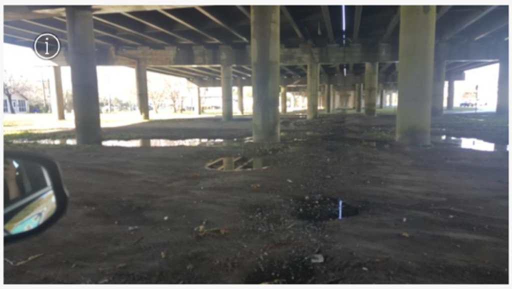

Left image: under I-26 bridge facing South | Right image: under I-26 bridge facing North

On this page... EJ Profile... Results from outreach... Interactive map contents... Interactive map... Contact Information...

Interactive map contents...

What is the interactive map?

The interactive map available on this site contains data layers relevant to residents' concerns about flooding and the seawall. Users can freely access and interact with these data layers for their personal use.

How do I use the interactive map?

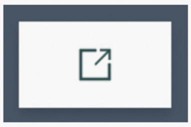

Screenshot of full screen button

1) Scroll to the "Interactive map" section to use on this site, or click the full screen button on the top right to open the map in a new tab.

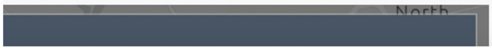

Screenshot of Legend button

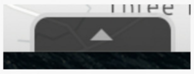

Screenshot of button for additional information

5) Some data layers like "Important places" contain additional information that can be viewed by clicking the grey arrow button at the bottom of the map.

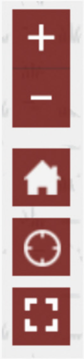

Screenshot of navigation buttons

6) Use the navigation buttons on the top left to zoom in and out, return to the map's original position, or zoom directly to your location. You can also click and drag directly on the map to move around.

7) Refer to the Data Layers section below for descriptions of all the layers available in the map.

On this page... EJ Profile... Results from outreach... Interactive map contents... Interactive map... Contact Information...

Data Layers

Data layer title - *Description*

Communities - *Locations of known communities in the Neck*

Flood locations - *Locations identified by residents that experience flooding*

Industrial facilities - *Locations of industrial facilities that have experienced hazardous releases, according to the EPA's Toxic Release Inventory*

Important places - *Locations of important businesses/areas/places in the Neck, identified by residents.*

Neck area - *Approximate location of the Neck of the Charleston peninsula*

NHC storm surge projections - *Depict storm surge potential in a high tide scenario for categories 1-5 storms. Developed by the National Hurricane Center (NHC)*

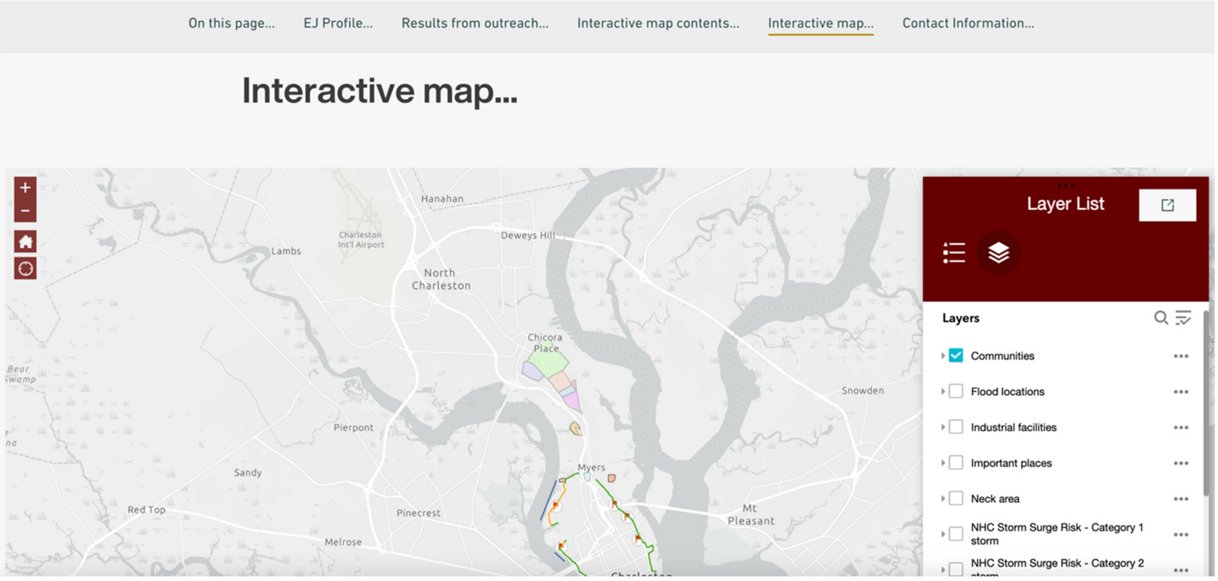

For access to the project information and any questions, please contact the Lowcountry Alliance for Model Communities (Lamcinfo18@gmail.com) or Judy Taylor (taylorj4@g.cofc.edu or jtaylor8968@gmail.com).
